# FEEDBACK OF RESEARCH FINDINGS FOR VACCINE TRIALS: EXPERIENCES FROM TWO MALARIA VACCINE TRIALS INVOLVING HEALTHY CHILDREN ON THE KENYAN COAST

**DOI:** 10.1111/dewb.12010

**Published:** 2013-02-21

**Authors:** Caroline Gikonyo, Dorcas Kamuya, Bibi Mbete, Patricia Njuguna, Ally Olotu, Philip Bejon, Vicki Marsh, Sassy Molyneux

**Keywords:** research ethics, sub-Saharan Africa, empirical ethics, clinical trials, benefit sharing

## Abstract

Internationally, calls for feedback of findings to be made an ‘ethical imperative’ or mandatory have been met with both strong support and opposition. Challenges include differences in issues by type of study and context, disentangling between aggregate and individual study results, and inadequate empirical evidence on which to draw. In this paper we present data from observations and interviews with key stakeholders involved in feeding back aggregate study findings for two Phase II malaria vaccine trials among children under the age of 5 years old on the Kenyan Coast. In our setting, feeding back of aggregate findings was an appreciated set of activities. The inclusion of individual results was important from the point of view of both participants and researchers, to reassure participants of trial safety, and to ensure that positive results were not over-interpreted and that individual level issues around blinding and control were clarified. Feedback sessions also offered an opportunity to re-evaluate and re-negotiate trial relationships and benefits, with potentially important implications for perceptions of and involvement in follow-up work for the trials and in future research. We found that feedback of findings is a complex but key step in a continuing set of social interactions between community members and research staff (particularly field staff who work at the interface with communities), and among community members themselves; a step which needs careful planning from the outset. We agree with others that individual and aggregate results need to be considered separately, and that for individual results, both the nature and value of the information, and the context, including social relationships, need to be taken into account.

## Background

Current research guidelines recommend the provision of aggregate results to research participants as good ethical practice.^1^ Internationally, calls for feedback of findings to be made an ‘ethical imperative’ or mandatory have met with both strong support and opposition.^2^ A fundamental challenge in discussions on researchers’ responsibilities and obligations, on participant preferences, and on the potential effects of feedback of findings, has been a lack of distinction between aggregate study results (representing synthesised data and conclusions from a group of research participants), and individual study results (representing distinct items of data collected from or about individual participants).^3^ Guidelines for feedback of findings that consider these differences are currently being developed, amended and critiqued.^4^

Across both types of results, the main overall arguments for providing feedback to participants include showing respect for participants by not treating them as a means to an end, and allowing participants to use the results to make positive changes to their lifestyle and to impact on their current and future health. Providing feedback of research findings also provides an activity that allows the participant to complete their involvement in the research, and potentially enhances trust in the researcher/research team, clinicians and the research process in general. The latter has the potential to improve the general perception of research in the community, and to demystify the research process to the public, which could in turn help increase uptake of participation in future research.

Arguments against, or challenges with, providing feedback of both individual and general research findings include: the possibility of causing distress to the participant when the results are negative or have the potential to cause emotional harm now or in the future; ‘survivor guilt’ for those assigned to the superior arm of the study; the potential for participants to not want results; potential future discrimination for participants in terms of employment and insurance; lack of general standards on feedback as different studies require different feedback mechanisms; and the feedback process itself being an additional research process with resource implications. Researchers have reported being particularly wary of providing inconclusive and potentially misleading information. Further practical challenges include the difficulty of developing lay versions of key information, the time it takes to have ‘a result’ in many studies, and the difficulty of tracking down some sample donors.

Even amongst those advocating for feedback as an imperative, there are divergent views on best practices regarding what the communication should contain, and on whether to give individual or aggregate results or both. Also not agreed is how much information should be given, when it should be given, who should give information, and how feedback should be integrated into the whole research process. What is agreed is that the process is far from straightforward, and that there can be challenges beyond the control of the research team. It is recognised that caution is required, especially when the results are negative or have the potential to harm the participant or others now or in the future. Also agreed is that there is currently inadequate empirical evidence on which to draw in debates on appropriate approaches to feedback. Research on feedback to date has been conducted in developed countries, illustrating a particular gap in voices and experiences from developing countries.

If and how to feedback results to paticipants, and researchers’ obligations, arguably depend on whether results are aggregate or individual,^5^ and on the nature and context of the research.^6^ In this paper we document the strategies developed to feedback aggregate results to participants in a particular type of research: two Phase 2 malaria vaccine trials involving healthy children aged less than five years old, each of which was conducted over a period of several years. The trials were conducted by a large research institution with several decades of experience of research in and around the low income rural communities on the coast of Kenya that were involved in the studies.

Both trials employed community-based fieldworkers to assist with the awareness raising, recruitment, surveillance and follow up processes of the wider trial, and more specifically with the feedback of agregate and individual findings at the end of the trials. In both trials, participants were followed up and treated free of charge for all acute illnesses identified over the course of trials, and referred for further treatment and support for chronic illnesses. Treatment and support of acute and chronic illnesses included feedback and discussion of results as part of clinical care.

In this paper we focus on feedback of aggregate findings at the end of the trials. As will be shown, the approach taken to feeding back findings was based on participant and community preferences, and therefore also included some feedback of indivdiual information. We describe the feedback strategies adopted at the end of main trial periods, and fieldworker and parent reactions to the results and to how they were delivered. We draw on the findings to consider the practical and ethical implications for similar future trials conducted in such contexts by established long-term research programmes.

## Methods

We focus on two trials – FFM ME-TRAP and RTS,S/AS01, which had 447 and 405 participants in Kenya respectively (Table 1). The first had ‘negative’ findings (vaccine not efficacious in preventing clinical malaria) and the second ‘positive’ findings (vaccine efficacious), with the latter leading on to the current on-going RTSS phase III trial. Both trials were double-blinded randomised trials, using anti-rabies vaccine as the control, with detailed community engagement plans, including feedback to participants.

**Table 1:** Summary of the FFM ME-TRAP and RTS,S/ASO1E studies^7^,^8^

	FFM ME-TRAP Study	RTS,S/ASO1E Study
Location	Junju location, Kilifi district (Kenyan Coast)	Kenya and Tanzania.
		We focus on Kenyan participants, in Pingilikani and Junju locations, Kilifi district
Participants	405 healthy children aged 1–6 years	447 healthy children aged 5–17 months
Timing	– 1 year with an 11 month follow up period after vaccination	– 14 months with an 8 month follow-up period before releasing first results
	– February 2005 to February 2006	– March 2007 to April 2008
	– Monitoring continued in a follow up study	– Monitoring continued in a follow up study
Key findings	– Vaccine safe but not efficacious against clinical malaria	– Vaccine safe and efficacy 53% against clinical malaria

In Kenya, the malaria vaccine trials were conducted by the KEMRI-Wellcome Trust Research programme, which has had a long interest in community views and recommendations. Members of the Health Systems and Social Science research group (HSSR) conducted unstructured observations of the development of research findings messages and strategies (CG, BM, and SM), followed by structured observations of community based feedback meetings for FFM ME-TRAP (n = 6; observed by CG) and RTS,S/AS01_E_ (n = 14; BM). The latter included observations of attendance, information given, non-verbal and verbals reactions to key messages, and time taken.

For FFM ME-TRAP, observations were supplemented by interviews with fieldworkers, parents of participating children, community members not involved in the trial, and trial staff (n = 13 FGDs and 4 IDIs). For RTS,S/AS01_E,_ observations were supplemented by documentation of a meeting between twenty three fieldworkers the day after parents’ feedback meetings (n = 23 fieldworkers; BM).

All interviews were digitally recorded and later transcribed and – where necessary – translated. Data were managed by CG using NVivo, and by BM using Microsoft word, and were analysed using basic summary tables organised around key themes.

The social science work in this study was approved for science and ethics at the institutional and national level (SCC protocol no. 1463).

## Findings

Following a description of message development and content, and delivery of key messages, for both trials, we summarise reactions and recommendations first to the end of trial results, and then to the feedback process followed by the trial teams to deliver those results.

### Message development and content

Both trial teams drew on recommendations from parents of participating children, the local dispensary health committee, researchers at the KEMRI Centre, and study fieldworkers when preparing feedback sessions. For the FFM ME-TRAP study, this process was formalised through a social science sub-study to the main trial.^9^ This sub-study illustrated that the inter-personal interactions and relationships between researchers and community members, and within the community, played a critical role in participants’ perceptions of trials, their decisions to consent or withdraw, and their advice to researchers on study practicalities and information to feedback at the end of the trial. Specifically there had been concerns that non-participants in the trial were spreading rumours about the dangers of the trial to children, including that blood was being taken by researchers for dubious purposes, and that this would eventually lead to children in the study dying. These relations contributed to participants’ parents recommending during the trial that:

1. the success of the vaccine at end of study should be ‘the first thing’ that is fed back;
2. participants should receive some form of recognition from the principal investigator or KEMRI for ‘hanging in there’ against all odds; for their contribution to that success; with suggestions including a party, and gifts.
3. the relationship between researchers and study participants should not be suddenly cut-off after the trial; that there should be some form of on-going reciprocity; and
4. there should be separate meetings for participants and general community, with any negative results kept secret from non-participants.^10^

Recognition of the above concerns, and of parents’ priorities generally, contributed to an emphasis in feedback plans on individual child status results (for example number of times the child had been unwell, including with malaria, and the haemoglobin (hb) status of the child over time) as well as overall trial findings for the FFM ME-TRAP study. This was in order to reassure parents of the child's own health status over the course of the trial despite the overall negative trial findings (Table 2). Also included in the general key messages was: information on what next, including continued follow-up and the introduction of another trial in the area; reasons why children's health overall had improved; a farewell and thanks from the researcher overseeing the main trial; and information that rabies vaccines had been donated to the local dispensary for use by any needy community member. Other information covered in individual feedback sessions was illnesses observed and treated.

**Table 2:** Key messages given during the FFM ME-TRAP and RTS,S/ASO1E studies

	FFM ME-TRAP Study	RTS,S/ASO1E Study
Broader/contextual information	Recap of study's aims and methods	– Definition of malaria and explanation of the health problems it causes
		– Recap of study's aims and methods
		– Frequently asked questions
Trial results	– Vaccine's inefficacy & safety	– Vaccine found to have 53% efficacy (ie ‘out of every 100 children vaccinated with RTS,S about half were protected from getting clinical malaria’)
	– Few side effects encountered	
		– in preventing against malaria therefore it is promising and requires further investigation on a larger scale and over a longer period
		– Vaccine's safety
Individual results	– Individual children's results explained to each parent by fieldworkers or researcher at the end of the meeting	– Not given yet – will come at the end of follow-up period
What next	– Continuity of follow ups, but with changes in study team, and need to go to dispensary for treatment rather than visits in homesteads (although treatment provision still supported by the study)	– Follow up period to continue once (ethical) approval is received
		– Reminder to continue using mosquito bed-nets as the vaccine was still under trial
		– Continuation of surveillance

For the RTSS trial, a priority was to present aggregate trial results to study participants before they appeared in an international publication, and the national media, but timed to ensure that results did not leak out to media in advance of planned press releases. The latter was based on an embargo from a journal. Individual results (specifically which trial arm the child was in) were not given out together with the general trial results, because of the importance of continued blinded follow-up of children. The focus of the aggregate results sessions was on the 53% efficacy, and on showing that while this was generally considered a positive message, malaria preventative measures were still essential for all. Individual results will be given on completion of the follow-up.

Summary information sheets outlining the key overall study results were prepared in Kiswahili and English for both trials.

### Delivery of key messages

*FFM ME-TRAP*. Following a briefing meeting with fieldworkers, aggregate feedback meetings for the FFM ME-TRAP study were held in five villages over a three day period (n = 6 meetings; 40 minutes to 1 hour twenty minutes for each meeting). Both parents were invited by fieldworkers to meetings in their own villages, but in practice relatively few of the 15–43 parents who attended each meeting were fathers. The meetings were led by the principal investigator (PI), supported by fieldworkers and the chairman of the local dispensary health committee. Following general information and discussion with all parents present, leaflets with general trial results were distributed. Parents of each child were then given their child's individual test results (for example on number of malaria cases over the trial), also summarised on paper. Fieldworkers later delivered results to non-attendees in their homes, including leaving a copy of the results sheets. The follow-up process took approximately one week.

*RTS,S/ASO1E*. 5 general study feedback meetings led by the PI and senior fieldworkers were all convened over two days, for the reasons outlined above. The format was similar to the FFM ME-TRAP process, although fieldworkers received the results for the first time together with the parents rather than before them. It was explained that individual children's results would not be released until a follow up study for which ethical approval was being sought. The importance of remaining blinded to trial arm was discussed. Information sheets were not distributed at these meetings primarily because of concerns that the data might be circulated in advance of the media discussion, but also because of doubts about the value of the printed material, and even worries that the key messages might be misinterpreted when read in a setting where they could not be discussed. Fieldworkers later delivered aggregate results verbally to non-attendees in their homes.

In both studies, fieldworkers invited parents to the feedback meetings, attended feedback meetings and assisted with interpretation at the meetings, and delivered results to parents who had not attended the meetings. They also followed up parents informally in their homes and in day to day interactions in villages to find out what concerns/questions they had after receiving the results.

### Overall reactions to the study results

The key overall difference between the two trials was disappointment with the news of the FFM ME-TRAP vaccine's inefficacy (something which emerged in discussions and interviews more than at the feedback meetings), contrasting with excitement to the news of the RTS,S/ASO1E vaccine's safety and apparent efficacy. Nevertheless the level of disappointment for ME-TRAP was not as great as expected. It appeared that many parents were either not convinced of the results, or believed that those results were irrelevant, given their own child's improvement:

So they are saying it didn't succeed, but I am saying it succeeded because I can finish 3 months before my child gets sick, [and since I joined the study] I forgot about going to the hospital. So whoever knows much is the one who says it didn't succeed, but on my side, I see it working because I had problems [before the trial] … (Father, FFM ME-TRAP study)

Less commonly, parents in the FFM ME-TRAP expressed concerns over their child's future health, saying that they were worried about the new research team and requesting for continued contact with the research team that they already knew. Occasionally, parents indicated that disappointment might lead some parents to withdraw their children.

For the RTS,S/AS01E feedback meeting, several parents wondered why their children should continue using bed nets if the vaccine had been found to be effective. This might have been linked to some confusion of what the key results actually meant, not only among the participants’ parents but also among field staff:

Let me say this, (pause) I am saying this on behalf of many of us. If we, the fieldworkers were not able to grasp the concept of how the 53% protection was arrived at, then we highly doubt if [the] majority of the parents understood it (laughter from other fieldworkers). Knowing the low literacy levels of the parents and the technical explanations that were given, to be honest, [the] majority of these parents did not grasp the concept. (RTS,S/AS01E fieldworker).

Other indications of parents not comprehending or believing the key messages were some parents describing both the malaria vaccines and rabies vaccine as on trial; leading to some FFM ME-TRAP parents reporting that the rabies vaccine had also ‘failed’. For RTSS, the reason why the individual children's results needed to be held back until the end of the follow up period was unclear to some parents.

Overall there were similarities across the two trials in what parents were most interested in finding out about and in what they most appreciated (Table 3). There was an interest in what benefits and support individual children and families would continue to get, whether those in the trial would receive the vaccine they had not yet received, and (for RTSS) whether all children in Kenya would now receive the vaccine. Parents appreciated the continuation of medical services and cessation of sample taking for research purposes. For the FFM ME-TRAP study, parents appreciated having received both individual children's and aggregate results, the continued employment of fieldworkers from their communities, and the researcher having come to say goodbye. The continuation of medical services also reportedly helped them save face in the community following the ‘failure’ of the vaccine trial, and assured them about the research team's motivation and continued support.

**Table 3:** Similarities in reactions to receiving results in both studies

• Parents were most interested in finding out:
◦ individual children's results/vaccine given rather than aggregate study results
◦ whether or not the study/study benefits would continue
◦ more about the follow up study
◦ whether those in the intervention arm would receive the rabies vaccine
• Appreciation for:
◦ continuation of study/study benefits
◦ end of sample-taking
◦ continued surveillance by the fieldworkers
•Difficulties in understanding study results:
◦ What it means when a vaccine is 53% effective (RTS,S/AS01E)
◦ Why malaria vaccine was ineffective and yet children's health improved (FFM ME-TRAP)
◦ False understanding by some that both vaccines – malaria and rabies – were under trial
•Requests for:
◦ children in each arm of the trial to receive the other vaccine/effective vaccine
◦ reciprocity from the researcher: party, gifts, new dispensary

Parents in both studies requested reciprocity as a reward for having co-operated with the study to the end, including for example farewell parties, gifts, and the upgrading of the local dispensary or building of new dispensaries. Of interest was that in the FFM ME-TRAP study, there was a negative reaction to news that rabies vaccines would be donated to the dispensary for use of anyone in need, with several parents vehemently protesting in feedback meetings (Box 1). This sense of participants owning the study benefits was even stronger in group discussions, with parents arguing that non-participants should not have access to the study-related benefits, and should not be given preference in participation in the upcoming study (since they had not ‘offered’ their children for the current study); and should not be given free malaria vaccines when the vaccine is finally developed.

**Box 1. Reactions by some parents in ME-TRAP to news that the rabies vaccine would be given to the dispensary as a benefit to all community members needing it; regardless of study participation**

“The rabies vaccine should be given to those who participated only but not to those that refused to participate. Even if a dog bites one, they shouldn't tell them there is the vaccine at the dispensary. They should go to Kilifi [hospital] because this vaccine is for those that participated!”.

“We do not accept! We do not accept it at all! And if you do so, we will withdraw completely from the study! We want to be vaccinated: us, our children, our husbands and even our dogs!”

“Maybe they [non-participants] are the ones that will be bitten by dogs and we will not get that vaccine …”

(Mothers, FFM ME-TRAP study)

### Community views and recommendations on how to feedback results

Receiving feedback was very much appreciated by parents, and for the first trial in particular it was appreciated that many of their ideas and recommendations had been included in the process and that the researcher had come to say ‘good-bye’. Across both trials however there were some concerns raised about the processes followed. Regarding, *amount of information and form it is given in,* some RTSS mothers attending the feedback sessions reported information overload, and concerns about not having received a leaflet:

How do you expect us to remember and deliver the same information you have given us to our spouses and other members of the family? (Mother, RTS,S/AS01E study)

We are used to taking written documents to our family. You should have given us our own copies of what you were reading to take home. (Mother, RTS,S/AS01E study)

Those not able to attend the meetings but who did receive a leaflet commented that it was an inadequate source of information on its own. News that the individual children's results in the RTS,S/AS01E study would not be released at that time were not well received by most parents:

You did promise to inform us of the results at the end of this study. You have now changed your mind. What guarantee do we have that you will not tell us the same story at the end of the next study? (laughter from the other parents) … (Father, RTS,S/AS01E study).

There were also concerns about *who was involved when*. That the trial findings were given to parents first was appreciated in both trials, but some queried the time taken between the end of active sample collection and receiving the feedback. Some dispensary health committee members and fieldworkers complained in RTSS about inadequate involvement in feedback planning, and not having received the results first.

### Withholding trial information from fathers and non-participants (FFM ME-TRAP)

Some mothers had apparently not informed their spouses or others about the study results, or about which particular arm of the trial their child was in. One reason appeared to be mothers being fearful of their spouse's reaction to information that the child had received the ‘failed vaccine’. This may have been linked to other gaps in information between mothers and husbands, including in details given out during study enrolment. It appeared that some mothers told their spouses about trial benefits and left out potential side effects, and that some even decided not to inform the father about the child's involvement at all. Another reason was a perception that the results should not be shared. This may have been the result of feedback sessions being held for participants only, and of individual results only being given out to a participant's parent because they are confidential. Confidential is often translated by research staff into local languages as ‘secret’. Finally, some mothers did not report results to non-participants to minimise embarrassment, mockery or new rumours resulting from the news of the vaccine being ineffective.

## Discussion

We have described the process used to feedback findings from two Phase II malaria vaccine trials involving children under the age of 5 years old on the Kenyan Coast, and participants’ parents reactions to the results and their delivery. Both trials were based in rural communities, and required a relatively intense relationship between research teams and participants over an extended period, in terms of children having been administered with an experimental (or control) vaccine, and regular blood sampling and health check-ups in dispensaries and in participants’ homes. Our findings are likely to be particularly relevant for such community-based trials in low-income settings, as opposed to hospital-based or genetics studies, or to studies involving less intense or long interactions between research teams and participants.

### Feeding back findings: complex but an opportunity

Overall our findings reflect those of others who report that research participants appreciate receiving aggregate results of trials that they have participated in.^11^ However, even for these relatively small trials, it was clear that feedback of findings is a complex process. This appreciation and complexity suggests that feedback of findings should be considered an intervention in its own right, which requires careful, rigorous and consultative planning right from the protocol development stage.^12^ Our research suggests that parents’ expectations of dissemination meetings are likely to include individual level information (including study arm and child's health status); and that parents’ hopes for and reactions to trial results will be based on concerns, expectations and tensions built up over the course of the study. This will only in part be based on information giving as part of a trial's wider community engagement processes. In our setting the feedback process was part of a continuing relationship, with the fieldworkers who came from and who continued to live in those communities being central players in that on-going relationship. The feedback sessions themselves appeared to be an important opportunity to re-explain, re-evaluate and re-negotiate trial relationships, processes and benefits; with potentially important implications for perceptions of and involvement in future research.

These findings have two important implications, discussed in turn below.

### Incorporating community priorities and concerns into feedback processes and messages

The development of specific messages will need to take into account the priorities and concerns of the participants or their parents, and of the key research and community members involved in the trial in the local setting. A challenge is that participant and community priorities may differ from those of researchers. For parents, personal observations of improvement in health,^13^ or about intra-community tensions and relations,^14^ may over-ride all other information. If researchers respond to parents' interest in detailed individual level information, there is a potential for community members to see the activity as primarily designed to understand and improve the health status of individual children, in turn possibly feeding into ‘therapeutic misconceptions’, or ‘diagnostic misconceptions’. This would have potential negative implications for the participants’ health, for example through a perception that the vaccine the child has received has the same level of efficacy as other routine vaccinations, and that malaria need no longer be a concern. Such interpretations may also impact on the validity of informed consent processes in future studies, through contributing to a view of the research centre as a good quality hospital, and a crowding out of research information through greater interest in and attention to health care benefits.^15^ While the latter is understandable in this context, of concern is where the research information, including risks, is not heard, or clouded over, by interest in benefits.

Regarding researchers responding to intra-community tensions generated through research activities, if and where these arise, a dilemma is what can be done to minimise rather than exacerbate those tensions. In both cases, information at the end of the trial might include both individual and overall study results, with individual information potentially important from the point of view of the participants, to reassure them of trial safety, and the research team, to ensure that positive results are not over-interpreted and that individual level issues around blinding and control are clarified. We would agree with others that individual and aggregate results need to be considered separately,^16^ and that for individual results, both the nature and value of the information, and the scope of entrustment involved in the research, the intensity and duration of interactions with participants, and the vulnerability and dependence of the study population,^17^ need to be taken into account when deciding if and what individual information should be given. This could be considered at the proposal development stage to allow adequate feedback mechanisms and resources.^18^

Regardless of what approach is taken, clear messages on what type of information will be given to whom, and at what stage, should be incorporated into community engagement strategies from the earliest possible stage. Failure to deliver on what are seen as promises can be undermining of appropriate trust relations, which are essential to both participants' perceived well-being and the success of trials. Messages for the feedback sessions themselves – both verbal and printed – are likely to need pre-testing and amendment in advance, and to be administered to both individuals and groups. Fieldworkers, given their key role at the interface with communities, and their own potential confusion, could be centrally involved in message development and delivery. This could be part of a careful training programme which also includes handling questions, concerns and expectations over time, and what issues to refer on and to whom.

### Feedback of findings as a key step in continuing social interactions

The second implication of our findings – linked to the first – is that in community-based studies in our settings, feedback of findings cannot be considered as once-off events delinked from previous relationships in the trial, or without future practical and ethical ramifications or implications. Careful consideration, with community representative inputs, of the benefits and risks that accrue to both individuals and the broader community, and strong community engagement plans, including informed consent processes that involve the father and mother wherever possible, potentially offer a good foundation for future feedback.

Our data also suggest that those community members and gate-keepers, including research centre staff, who are most likely to be visited for further information or advice once the trial is over, need to be included in feedback activities, and be equipped with adequate information to answer basic questions, and information on when and where to refer any major issues or concerns that arise in the weeks or months after the results have been formally presented. It is also important to consider from the outset of a trial that some of those who are turned to in the community once the trial ends may be losing some social and resource benefits towards the end of the trial; potentially even employment. For example community leaders may have gained some respect by community members for having allowed or even encouraged a trial with health care benefits into the area, and community members employed as trial fieldworkers may no longer be needed. Thus feedback sessions become settings in which not only might trial participants or their parents be re-explaining, re-evaluating and re-negotiating their perception of and relationship with trial teams, but also fieldworkers and other local players are doing the same. Simply recognizing and thanking those who have been central to trial's success in public, regardless of whether the trial findings were ‘positive’ or ‘negative’, might be appreciated in that context.

## Conclusion

We found that feedback of findings is a complex but key step in a continuing set of social interactions between community members and research staff (particularly fieldworkers), and among community members themselves. We concur with others in recommending that the feedback process needs careful consideration from the outset on a case by case basis. In our context, including some individual information at ‘the end’ of the trial appeared to be important. Firstly, participants had a strong interest in receiving individual information on their child's overall health status changes over the course of the trial, and the arm of the trial they were in. While preferences do not define fundamental obligations, they are consistent with ethical principles of respect for persons and beneficence, and can promote building trust and support in research. From the point of view of the participants, individual information was also important to reassure them of trial safety, and for the research team, was aimed at ensuring that positive results were not over-interpreted and that individual level issues around blinding and control were clarified. Whether these goals were achieved need further future research, in a carefully designed prospective study that follows participants over time, post receipt of results.

## References

[b1] Hede K Efforts To Communicate Clinical Trial Results to Patients Face Uphill Climb. Journal of the National Cancer Institute.

[b01] Moutel G (2005). Communication of pharmacogenetic research results to HIV-infected treated patients: standpoints of professionals and patients. Eur J Hum Genet.

[b02] Nuffield Council on Bioethics (2005). The ethics of research related to healthcare in developing countries.

[b003] World Medical Association (WMA) (2000). Ethical Principles for Medical Research Involving Human Subjects-Declaration of Helsinki.

[b2] Dixon-Woods M (2006). Receiving a summary of the results of a trial: qualitative study of participants’ views. Bmj.

[b03] Fernandez CV (2004). Considerations and costs of disclosing study findings to research participants. Cmaj.

[b04] Partridge AH, Winer EP (2002). Informing Clinical Trial Participants About Study Results. JAMA: The Journal of the American Medical Association.

[b05] Shalowitz DI, Miller FG (2008). Communicating the Results of Clinical Research to Participants: Attitudes, Practices, and Future Directions. PLoS medicine.

[b06] Wang L (2002). Researchers Push for Sharing of Trial Results with Participants. Journal of the National Cancer Institute.

[b4] Beskow LM, Burke W (2010). Offering Individual Genetic Research Results: Context Matters. Sci Transl Med.

[b07] Fabsitz RR (2010). Ethical and practical guidelines for reporting genetic research results to study participants: updated guidelines from a National Heart, Lung, and Blood Institute working group. Circ Cardiovasc Genet.

[b5] Clayton EW, Ross LF (2006). Implications of Disclosing Individual Results of Clinical Research. JAMA: The Journal of the American Medical Association.

[b08] Shalowitz, Miller

[b6] Beskow, Burke

[b9] Gikonyo C (2008). Taking social relationships seriously: lessons learned from the informed consent practices of a vaccine trial on the Kenyan Coast. Soc Sci Med.

[b09] Molyneux S (2007). Incorporating a quiz into informed consent processes: Qualitative study of participants’ reactions. Malaria Journal.

[b11] Fernandez

[b010] Hede

[b011] Partridge, Winer

[b012] Shalowitz, Miller

[b013] Wang

[b12] Dixon-Woods

[b014] Dorsey ER (2008). Communicating Clinical Trial Results to Research Participants. Archives of Neurology.

[b14] Marsh VM (2011). Working with Concepts: The Role of Community in International Collaborative Biomedical Research. Public Health Ethics.

[b15] Meltzer HL (2006). Undesirable Implications of Disclosing Individual Genetic Results to Research Participants. American Journal of Bioethics.

[b17] Belsky L, Richardson HS (2004). Medical researchers’ ancillary clinical care responsibilities. Bmj.

[b015] Beskow, Burke

